# MSIpixel: a fully automated pipeline for compound annotation and quantitation in mass spectrometry imaging experiments

**DOI:** 10.1093/bib/bbad463

**Published:** 2023-12-14

**Authors:** Lavinia Morosi, Matteo Miotto, Sara Timo, Sara Carloni, Eleonora Bruno, Marina Meroni, Elisabetta Menna, Simona Lodato, Maria Rescigno, Giuseppe Martano

**Affiliations:** IRCCS Humanitas Research Hospital, Via Manzoni 56, Rozzano (MI), Italy; IRCCS Humanitas Research Hospital, Via Manzoni 56, Rozzano (MI), Italy; IRCCS Humanitas Research Hospital, Via Manzoni 56, Rozzano (MI), Italy; IRCCS Humanitas Research Hospital, Via Manzoni 56, Rozzano (MI), Italy; Department of Biomedical Sciences, Humanitas University, Via R. Levi Montalcini 4, Pieve Emanuele (MI), Italy; Department of Experimental Oncology, Fondazione IRCCS Istituto Nazionale dei tumori di Milano. Via Venezian 1, Milan, Italy; Istituto di Ricerche Farmacologiche Mario Negri IRCCS, Department of Oncology, via Mario Negri 2, Milan, Italy; IRCCS Humanitas Research Hospital, Via Manzoni 56, Rozzano (MI), Italy; Institute of Neuroscience, National Research Council of Italy (CNR) c/o Humanitas Mirasole S.p.A, Via Manzoni 56, Rozzano (MI), Italy; IRCCS Humanitas Research Hospital, Via Manzoni 56, Rozzano (MI), Italy; Department of Biomedical Sciences, Humanitas University, Via R. Levi Montalcini 4, Pieve Emanuele (MI), Italy; IRCCS Humanitas Research Hospital, Via Manzoni 56, Rozzano (MI), Italy; Department of Biomedical Sciences, Humanitas University, Via R. Levi Montalcini 4, Pieve Emanuele (MI), Italy; IRCCS Humanitas Research Hospital, Via Manzoni 56, Rozzano (MI), Italy; Institute of Neuroscience, National Research Council of Italy (CNR) c/o Humanitas Mirasole S.p.A, Via Manzoni 56, Rozzano (MI), Italy

**Keywords:** MSIpixel, annotations, metabolomics, mass spectrometry imaging

## Abstract

Mass spectrometry imaging (MSI) is commonly used to map the spatial distribution of small molecules within complex biological matrices. One of the major challenges in imaging MS-based spatial metabolomics is molecular identification and metabolite annotation, to address this limitation, annotation is often complemented with parallel bulk LC-MS2-based metabolomics to confirm and validate identifications. Here we applied MSI method, utilizing data-dependent acquisition, to visualize and identify unknown molecules in a single instrument run. To reach this aim we developed MSIpixel, a fully automated pipeline for compound annotation and quantitation in MSI experiments. It overcomes challenges in molecular identification, and improving reliability and comprehensiveness in MSI-based spatial metabolomics.

## INTRODUCTION

Mass spectrometry imaging (MSI) is commonly used to map the spatial distribution of specific classes of biomolecules, such as drugs and metabolites, within complex biological matrices [1]. One of the most widely used ionization techniques for this type of analysis is matrix-assisted laser desorption/ionization (MALDI), which can achieve a resolution of a few μm per pixel[2]. MALDI-MSI has shown great potential in the field of metabolomics [3, 4] and is increasingly being applied directly to tissue samples to uncover metabolic changes associated with different pathologies and treatments [1, 5]. The major challenges in imaging MS-based spatial metabolomics are the molecular identification and metabolite annotation. The available approaches and software to face this problem are summarized in an excellent review by Baquer et al. [6]. The current state-of-the-art approach relies on determining the mass of the peaks of interest in the MS scan to assign chemical compositions, based solely on high mass resolution and accuracy of the instrumentation[7, 8]. Annotation is often complemented with parallel bulk LC-MS/MS-based metabolomics to confirm and validate identifications [9–11].

The combination of these methods has its own limitations, leading to annotation errors and restricting the number of detectable metabolites in MSI experiments. Two major issues arise when combining MSI and LC-MS/MS: (i) missing identifications in LC-MS/MS due to instrumental sensitivity for compounds with a limited spatial distribution, and (ii) the loss of identification due to differential affinity between the extraction solvent in LC-MS and the matrix desorption in MSI. Overall, identification in LC-MS/MS may not reflect MSI data accurately. Only a few pioneering MSI methods have been developed to allow multiple data acquisition types in a single instrument run, aiming to visualize and identify unknown molecules [12]. However, these methods often sacrifice spatial resolution and yield a relatively low number of identified metabolites/lipids [12]. Major limitations hindering the development of more comprehensive approaches stem from extrinsic factors such as instrumental sensitivity and slow scan rates, as well as intrinsic factors such as the lack of normally distributed signals within the samples. Fortunately, the latest MS instruments are more sensitive and faster than their predecessors, offering a wide range of acquisition options for designing MS experiments that can overcome the limits of MALDI-MSI. Data-dependent acquisition (DDA) methods provide several features, including the ability to define a dynamic exclusion criterion for recurrent signals, set a minimum intensity value for precursor selection for further MS2 analysis, and define sets of values to include or exclude as targets of interest. Together, these rules define a user based peak picking approach for generating MS2 spectra that can be used for features selection and annotation in MSI experiments.

In order to be able to analyzed MSI data with DDA we have developed MSIpixel, a fully automated pipeline that enables compound annotation and quantitation.

## MATERIAL AND METHODS

### Reagents

LC-MS grade acetonitrile, methanol and trifluoroacetic acid were purchased from Carlo Erba (Milan, Italy). α-Cyano-4- hydroxycinnamic (CHCA) was purchased from Sigma-Aldrich Co. (St. Louis, Missouri, USA). Deionized water was produced using a Milli-Q water purifying system (Millipore Corp., Bedford, MA, USA).

### Sample preparation

MPM487 is a malignant pleural mesothelioma xenograft with biphasic morphology established in our group from patient derived cell lines and maintained by serial transplantation in nude mice. Histological and immunohistochemical features reproduce its biphasic morphology [13].

Six- to eight-week-old female NCr-Nu/Nu mice (Envigo, Udine, Italy) were used. MPM487 tumor fragments were implanted subcutaneously in the right flank of nude mice. For sampling, mice were anesthetized by isoflurane and blood collected from the retro-orbital plexus into heparinized tubes. Tumors were explanted and immediately snap-frozen in liquid nitrogen and stored at −80°C until MSI analysis. Procedures involving animals and their care were conducted in accordance with the following laws, regulations, and policies governing the care and use of laboratory animals: Italian Governing Law (D.lgs 26/2014; Authorization n.19/2008-A issued March 6, 2008 by Ministry of Health); Mario Negri Institutional Regulations and Policies providing internal authorization for persons conducting animal experiments (Quality Management System Certificate – UNI EN ISO 9001:2008 – Reg. N° 6121); the NIH Guide for the Care and Use of Laboratory Animals (2011 edition) and EU directives and guidelines (EEC Council Directive 2010/63/UE) and in line with Guidelines for the welfare and use of animals in cancer research. Experimental protocols have been reviewed and approved by the IRFMN Animal Care and Use Committee (IACUC) that includes members “ad hoc” for ethical issues, and by the Italian Ministry of Health.

Frozen tissues were cut in 10 μm thick sections using a cryomicrotome (Leica Microsystems, Wetzler, Germany) at −20°C and mounted on indium tin oxide (ITO)-coated glass slides by standard thaw-mounting techniques. One section every 300 μm was cut starting from the central part of the tissue. The plate was dried in a vacuum drier at room temperature for 1 h and then sprayed with CHCA (10 mg mL-1, CH3CN 70% and TFA 0.1%) using a Sun Collect MALDI-sprayer (SunChrom, Friedrichsdorf Germany) with following spray protocol: Z-Axis 25 mm, 10 matrix layers, 2.5 bars (36 PSI) gas pressure, 600 mm/min spray speed, 2 mm line distance and variable flow rate (10; 20; 30; 40; 8 ×60 μL/min).

### MSI acquisition

An Orbitrap™ IQ-X™ Tribrid™ Mass Spectrometer (IQ-X) and an Orbitrap Exploris™ 120 Mass Spectrometer (Thermo Fisher Scientific Waltham, MA) both coupled with AP-MALDI-ng-UHR ion source (MassTech Inc., Columbia, MD) was used controlling the source with Target-ng software (MassTech Inc., Columbia, MD). Laser energy of 3.5%, 3kV voltage applied onto the plate. Tune and Xcalibur 4.3 software (Thermo Fisher Scientific Waltham, MA, USA) were used to control the mass spectrometer parameters with the following settings: ion transfer tube temperature of 300°C, positive ion voltage 2500 V and RF 70 were used unless otherwise specified.

For MSI experiments, constant speed raster motion was used with 10–100 μm spatial resolution and plate velocity dependent on scan time: with a 0.6 scan time, at 100 μm spatial resolution, plate velocity was set at 10 mm/sec; at 10 μm spatial resolution, plate velocity was set at 1 mm/sec Automatic gain control was set at 100% with 100 ms maximum injection time. The instrument was mass calibrated with FlexMix Calibration Solution using Auto-Ready system, combined with the Thermo Scientific™ EASY-IC™ (Internal Calibration). The acquisition method includes in a full scan acquisition with positive polarity, mass range of m/z 150–900, resolution of 120K and a DDA-MS/MS semiparallel (0.6 sec scan time on IQ-X) or sequential (TOP 4 on Exploris 120). Key details of DDA-MS/MS for both instruments are summarized in Table 1. Dynamic exclusion Exclusion list was generated using AcquireX on IQ-X and then added to Exploris 120 method. As regard the dynamic exclusion properties, a precursor is excluded after 2 times if occurs within 3 sec and the exclusion duration was set at 5400 sec for both instruments.

**Table 1:** Key details of DDA-MS2 acquisition methods

	**IQX**	**Exploris 120**
Isolation mode	Quadrupole	Quadrupole
Isolation window (m/z)	1	1
Activation type	CID	HCD
Collision energy mode	Fixed	Fixed
CID collision energy (%)	40	
CID activation time (ms)	10	
Detector type	Ion Trap	Orbitrap
Ion trap scan rate/resolution	Enhanced	30000
Scan range mode	Auto	Auto
AGC target (%)	100	100
Maximum injection time (ms)	Auto	Auto

Abbr. Collision induced dissociation (CID); Higher-energy collisional dissociation (HCD)

### Data analysis

Raw data acquired with Orbitrap Exploris 120 Mass Spectrometer and Orbitrap IQ-X Tribrid Mass Spectrometer were converted in mzML format using MSConverter from ProteoWizard [14]. MS2 spectral library was generated by extracting “MS-MS Spectra Files (XML) – Predicted” and “MS-MS Spectra Files (XML) – Experimental” and available on MSIpixel in the library section. Analysis was with a tolerance of 5 ppm without correcting for the total ion current (TIC). Partial least squares discriminant analysis (PLS-DA) was performed with Metaboanalyst 5.0 on the raw excel exported data without normalization, transformation or scaling.

### Graphic User Interface (GUI) and modules

MSIpixel was developed in Python using pymzml [15]as mzML reader, the graphic user interface was builded using PyQt5, with Seaborn [16] and matplotlib [17]for data visualization. The executable was compiled with Pyinstaller. Modules used for data processing include Scipy [18], Numpy [19]. The software is available at https://github.com/gmartano/MSIpixel

### WorkflowMS/MS quality assessment in MSIpixel

MSIpixel is designed for analyze single raw files and required three inputs i.e. the mzML file, the relative XML file and a folder containing one or more library intended to be used for the analysis. Three parameters can be set-up by the user by selecting parallel acquisition method, normalization against the TIC, and the maximum part per million (ppm) error for data mining.

**Figure 1:**
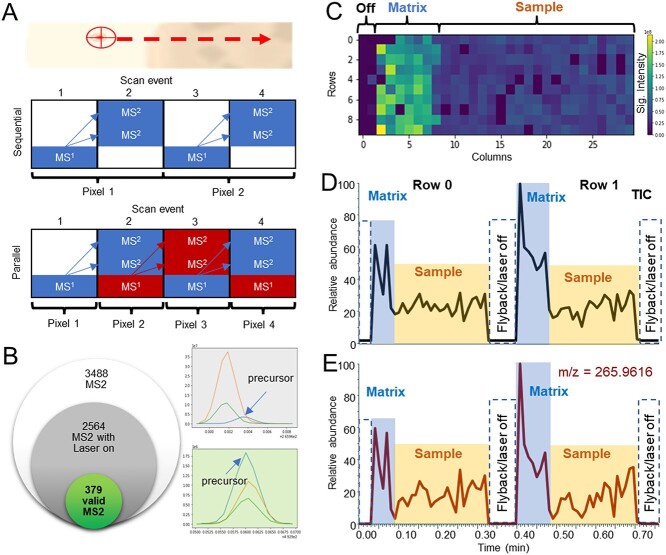
**DDA principles and matrix effect in MSI.** (**A**) Graphical representation of sequential or parallel MS and MS2 acquisition during MSI experiment. (**B**) Venn diagram with different quality of MS2 spectra from HCCA_MS2 file reporting total MS2, the number of MS2 with active laser and number of MS2 passing the quality test with 5 ppm tolerance. (**C**) Total ion map report TIC intensity for each pixel from HCCA_MS2 file. (**D**) TIC from the HCCA_MS2 chromatogram. (**E**) Extracted ion chromatogram of mass 265.96 which is characteristic of the matrix.

The initial feature selection is based on the all MS2 events recorder by the mass spectrometer. Depending on the instrumental architecture, MS2 spectra can be acquired sequentially with full scan spectra, which increases the scan time per pixel, or in MS instruments with multiple detectors, parallel acquisition allows quasi-simultaneous acquisition of both full scan and MS2 spectra (Figure 1A). In both modes, precursor selection and isolation for MS2 spectra occur in separate scan events, necessitating a quality assessment of the acquired MS2 data. To filter out non-valid MS2 spectra, we used two criteria: (i) the laser must be active, and (ii) the selected precursor must show consistency within a given error tolerance expressed in ppm across two consecutive scan events for parallel acquisition, or one scan event for sequential acquisition, following the scan in which the precursor was chosen (Figure 1B).

In MSI experiments, samples of interest are mounted on a conductive surface and coated with a matrix to facilitate the desorption and ionization of the compounds of interest. The matrix is irradiated with a laser, and the generated ions enter the mass spectrometer, contributing to the TIC. The matrix's contribution to the TIC represents a significant fraction of the ions reaching the mass spectrometer, and the TIC intensity is generally higher outside the sample region, as the biological samples act as insulating materials, reducing surface conductance (Figure 1C and D). Signals from the matrix also contribute to the TIC in the sample region (Figure 1E), but these signals are typically not relevant to the tissue being analyzed. Therefore, it is recommended to exclude matrix signals before acquisition to avoid wasting valid MS2 spectra.

### Chemical formula and MS2 score

**Figure 2:**
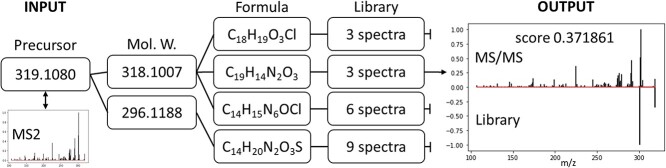
Pipeline summarizing the annotation steps between the precursor selection and the annotation against a given library.

From MS2 spectra that pass the initial quality check, MSIpixel extracts the list of all the possible precursor ions that were selected for MS2 scan. For each precursor, we retrieve all possible neutral molecular weight taking into account most common ions in MALDI-MS experiments i.e. [M+H]^+^ and [M+Na]^+^ for positive ionization mode and [M-H]^-^ and [M+Cl]^-^ for negative ionization mode. Calculated molecular weight and calculated exact mass from each compound in the libraries are matched. For each match between empiric and calculated exact masses has an error tolerance calculated in ppm. Ppm value is defined by the user as parameter in the analysis. After matching, each MS2 spectra can be now bounded to different MS2 spectra from libraries, as within the same ppm tolerance, many exact mass with different ion adduct or event within the same adduct but different formula, may fall into the given ppm tolerance. Moreover, within the same adduct and same formula, we may have several isomers or multiple times the same compound present in the libraries with different collision energy. As a result, for single spectra, we may end up having hundreds of matches. In the last stage of annotation, each matched is compared by computing the cosine similarity between the empiric MS2 spectra and the MS2 spectra retrieve from the library match, and only the highest score is retain (Figure 2).

### Target exclusion selection

For rapidly generating the target exclusion list, we used the AcquireX method by acquiring a single line for 30 sec inside the exclusion region. The AcquireX® method automatically updates the exclusion list of the DDA method. The method is edited by update the analysis duration both method and the exclusion list and used for acquiring the region of interest (ROI). This step is required to increase annotation coverage as most of the TIC composition is given by the matrix, the presence of the exclusion list is pivotal to maximized meaningful DDA isolation by the instrument. The exclusion region can be any area at least 2 mm outside the ROI. This distance may vary based on the solvent, matrix, and setup in used for spraying the matrix, and it is related to the maximum signal delocalization distance observed empirically+

**Figure 3:**
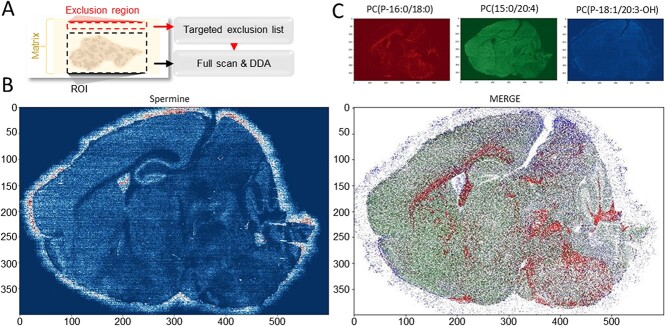
**ROI selection and compound delocalization.** (**A**) Workflow scheme. (**B**) An example of metabolite (e.g. spermine) delocalization from brain tissue slice that can affect the exclusion list generation. (**C**) EIM of representative identified metabolites: Left panel PC(P-16:0/18:0), central panel PC(15:0/20:4), right panel PC(P-18:1/20:3-OH), merge.

To preserve valid MS2 spectra for meaningful compound annotation, a target mass exclusion can be added in the method (Figure 3A). This option, available in all high-resolution mass spectrometers suitable for DDA, is a list of masses which are ignored when the instrument is selecting the precursors to be isolated for MS2 experiments. Many automated pipelines are available nowadays thus reducing to a few minutes the time required for the generation of the exclusion list of masses. In MSI experiments, the region acquired for generating the exclusion list influences the outcome of the analysis. Metabolites may show lateral desorption or delocalization (Figure 3B) and therefore choosing an exclusion region too close to the tissue may include masses that belong to the sample and prevent their annotation due to the lack of MS2 spectra. On the other hand, small molecules display a broad range of chemical physical properties, and even within the same class of compounds, not all the metabolites will delocalize in the chosen matrix (Figure 3C). For this reason, generating the exclusion list nearby the tissue will allows to focus on the identification of compound with highly spatial coherence.

### Comparison of parallel and sequential DDA in MSI

In this study, we evaluated the performance of MSIpixel using both parallel and sequential acquisition approaches. We acquired MSI data from a tumor tissue slice coated with HCCA (α-cyano-4-hydroxycinnamic acid). The spatial resolution for MSI was set at 100 μm per pixel, with the laser moving horizontally. It should be noted that with horizontal motion, the pixel size on the x-axis can be synchronized by adjusting the laser speed according to the instrument scan rate. However, on the y-axis, the ablated matrix remains within the laser diameter, which was ~5 μm in our case. This allowed us to scan the same tissue twice, acquiring the second MSI between lines and utilizing a region of intact matrix.

For parallel acquisition, we used the IQ-X instrument, while sequential acquisition was performed using the Exploris 120. In both instruments, the MS1 scan was conducted at a resolution of 120,000 in the Orbitrap, while the MS2 scan was performed in the Ion trap for IQ-X and in the Orbitrap for Exploris 120. The full scan cycle was set to 0.6 sec per pixel in both cases.

**Figure 4:**
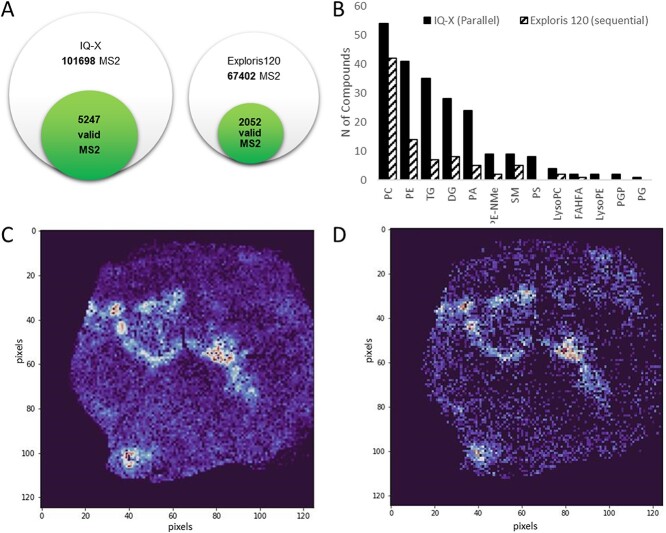
**Parallel and sequential DDA.** (**A**) Venn diagram of the identified metabolites with the two different acquisition methods. (**B**) Number of identified lipids divided in the different classes. Spatial distribution of a sphingomyelin compound a sphingomyelin compound detect with IQ-X (**C**) or Exploris 120 (**D**).

As expected, parallel acquisition resulted in a higher number of MS2 events, leading to a greater number of valid MS2 spectra based on the quality rules we previously defined (Figure 4A). In addition, parallel acquisition provided a broader coverage of lipid species that could be identified from a single experiment (Figure 4B).

Notably, another critical factor influencing both the quality of extracted ion maps (EIMs) of compounds and their annotation is sensitivity. We compared the EIMs of a sphingomyelin compound identified by both analyses (Figure 4C and D). It is evident that a few pixels fall below the detection limit of the IQ-X compared with the Exploris instrument. The variation in sensitivity can be attributed to instrumental differences. However, it is important to consider that instruments with lower sensitivity are less likely to generate valid spectra, as the selection may occur before reaching a pixel below the detection limits. Consequently, this limitation also impacts the identification coverage.

### Annotation comparison between high-resolution and MS2 based approach

**Figure 5:**
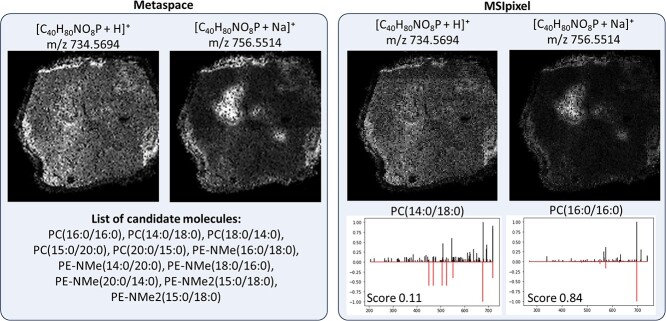
Comparison between identification at full scan and MS2 level.

Based on the available software for MSI analysis, for which an exhaustive list can be found in a recent review from Baquer et al, we chose Metaspace as it required minimal user input and accept similar libraries i.e. Human Metabolome Database (HMDB), as the one we used for testing MSIpixel functionality. Briefly, an imzML file was generated from the original XML and mzML file with MT imzML Converter (MassTech) by setting the MSn filter equal to 1. This generates a canonical high-resolution imzML typically used for this type of analysis. In order to compare the analysis, we used identical settings as in MSIpixel i.e. used HMDB library, ppm error of 5 ppm. Metaspace was able to discover 25 features which can be inspected on Metaspace website (https://metaspace2020.eu/annotations?ds=2023-10-02_14h56m07s). All discovered chemical formulas and adducts were present in the analysis carried out with MSIpixel. Notably, when we compared adducts for the same chemical structure, we could appreciate that while for Metaspace adducts are clustered within the same annotation, in MSIpixel we could find different annotations. As for the case of phosphatidylcholine 32:0 (Figure 5) which has two acyl groups with a total length of 32 carbons and 0 unsaturation, we could observe that H+ and Na+ adducts display huge differences in their spatial distribution. This is due to the fact that, based on the used matrix and the different chemistry of PCs within this group, not all the isomers will equally ionized with both adducts. This preferential ionization can be appreciated in particular for the Na+ adduct showing a MS2 spectra that largely recapitulate the compound PC(16:0/16:0) while the H+ adduct is a combination of different isomers, thus causing a low MS2 score, that including PC(14:0/18:0) which is likely the most represented compound for this adduct.

## CONCLUSION

In this study, we developed MSIpixel, a fully automated pipeline for compound annotation and quantitation from MSI-DDA raw data. We addressed the challenges associated with molecular identification and metabolite annotation in imaging MS-based spatial metabolomics, without the need of LC-MS/MS analysis. Starting from MS2 spectra gave us the possibility to maximize the number of initial features and avoid loss of informative fragmentation spectra. The quality assessment of MS2 fragmentation spectra was crucial point for reliable compound annotation. We implemented criteria to filter out non-valid MS2 spectra, considering the laser activity and the consistency of selected precursors within a given error tolerance. Furthermore, we emphasized the importance of excluding matrix signals before acquisition to avoid wasting valid MS2 spectra. By comparing parallel and sequential acquisition approaches in MSI, we evaluated the performance of MSIpixel. Our results demonstrated that parallel acquisition provided a higher number of MS2 events and broader coverage of lipid species compared with sequential acquisition. By comparing the results from canonical high resolution annotation with MS2 annotation, we showed that differential spatial distribution of adducts can be now explained due to differential chemical preferences in forming adducts. Notably, those differences can be appreciated only if MS2 is acquired within MSI analysis while will be lost when LC-MS approaches are combined for identification. However, this approach highlights also the major limit in the current identification approach. MS2 spectra represented by more than one chemical compound will result in an annotation with poor MS2 score. Novel evaluation methods that enable in silico prediction of mixed spectra may enhance our ability to discriminate between isobaric compounds and their spatial representation.

Instrumental sensitivity played a significant role in the quality of EIM and compound annotation, as the difference in sensitivity can limit the generation of valid spectra and restrict the identification coverage of metabolites and lipids.

Overall, MSIpixel presents a promising approach for compound annotation and quantitation in MSI-DDA experiments. By addressing the challenges associated with molecular identification, it can enhance the reliability and comprehensiveness of MSI-based spatial metabolomics studies.

**Key Points**

- The manuscript presents MSIpixel, an automated pipeline for compound annotation and quantitation in MSI experiments, addressing challenges in molecular identification and improving reliability in spatial metabolomics.
- The pipeline overcame the limitations associated with MALDI-MSI and bulk LC-MS2-based metabolomics combination, such as instrumental sensitivity and differential affinity between extraction solvent and matrix desorption.
- MSIpixel utilizes DDA resulting in improved compound annotation and a higher number of identified metabolites/lipids in MSI experiments. The pipeline is implemented in Python and is available as an open-source software.

## Data Availability

Data available on request. The software is available at https://github.com/gmartano/MSIpixel.

## Notes

**Lavinia Morosi** is staff scientist and co-head of the Metabolomics Unit at Humanitas Research Hospital.

**Matteo Miotto** is a bionformatician in the Developmental Neurobiology Lab at Humanitas Research Hospital.

**Sara Timo** is a technitian of the Metabolomics Unit at Humanitas Research Hospital.

**Sara Carloni **is an Assistant Professor at the Department of Biomedical Sciences, Humanitas University.

**Eleonora Bruno** is a researcher at Fondazione IRCCS Istituto Nazionale dei Tumori di Milano.

**Marina Meroni** is Ph.D. candidate at Mario Negri Institute.

**Elisabetta Menna** is researcher at Institute of Neuroscience, CNR.

**Simona Lodato** is Associate Professor at Humanitas University, principal investigator of the Neurodevelopmental Biology Lab at Humanitas Research Hospital.

**Maria Rescigno** is the Vice Rector and Delegate for Research at Humanitas University.

**Giuseppe Martano** is a researcher at institute of Neuroscience, CNR and co-head of the Metabolomics Unit at Humanitas Research Hospital.
